# The siRNA targeted to *mdr1b *and *mdr1a *mRNAs *in vivo *sensitizes murine lymphosarcoma to chemotherapy

**DOI:** 10.1186/1471-2407-10-204

**Published:** 2010-05-14

**Authors:** Olga A Patutina, Nadezda L Mironova, Nelly A Popova, Vasily I Kaledin, Valery P Nikolin, Valentin V Vlassov, Marina A Zenkova

**Affiliations:** 1 Institute of Chemical Biology and Fundamental Medicine, Siberian Branch, Russian Academy of Sciences, Lavrentiev av. 8, Novosibirsk, 630090 Russia; 2 Institute of Cytology and Genetics, Siberian Branch, Russian Academy of Sciences, Lavrentiev av. 10, Novosibirsk, 630090 Russia

## Abstract

**Background:**

One of the main obstacles for successful cancer polychemotherapy is multiple drug resistance phenotype (MDR) acquired by tumor cells. Currently, RNA interference represents a perspective strategy to overcome MDR via silencing the genes involved in development of this deleterious phenotype (genes of ABC transporters, antiapoptotic genes, etc.).

**Methods:**

In this study, we used the siRNAs targeted to *mdr1b, mdr1a*, and *bcl-2 *mRNAs to reverse the MDR of tumors and increase tumor sensitivity to chemotherapeutics. The therapy consisting in *ex vivo *or *in vivo *application of mdr1b/1a siRNA followed by cyclophosphamide administration was studied in the mice bearing RLS_40 _lymphosarcoma, displaying high resistance to a wide range of cytostatics.

**Results:**

Our data show that a single application of mdr1b/1a siRNA followed by treatment with conventionally used cytostatics results in more than threefold decrease in tumor size as compared with the control animals receiving only cytostatics.

**Conclusions:**

In perspective, mdr1b/1a siRNA may become a well-reasoned adjuvant tool in the therapy of MDR malignancies.

## Background

A substantial progress in the study of cancer development was achieved during last decades. However, mortality from these diseases still remains high despite the appearance of new highly efficient therapeutics and new polychemotherapy programs. One of the main obstacles for successful polychemotherapy of cancer diseases is the development of multiple drug resistance (MDR) phenotype, the resistance to a wide range of chemotherapeutic agents, acquired by cancer cells [1].

The primary cause of MDR phenotype is attributed to an overexpression of some members of a highly conserved gene family (from bacteria and barley to man) of transmembrane ATP-binding cassette proteins, referred to as the ABC transporters superfamily [2-4]. The human *MDR1 (ABCB1) *gene and two mouse genes, *mdr1b *and *mdr1a *(*Abcb1b *and *Abcb1a)*, that belong to subfamily B of ABC-transporters encode P-glycoprotein (Pgp), involved in active ATP-dependent transport of cytotoxic agents out of the cells [5-7]. Being a complex process, MDR arises also from the expression abnormalities of other genes, namely, the genes encoding pro- and antiapoptotic factors p53, Bax, Bad, and Bcl-2 [5-10]; genes of the glutathione pathway [2,4]; and genes encoding topoisomerases [3,4,11]. However, the most frequent cause of the MDR phenotype development in many human and rodent tumors is an increased level of Pgp [12-14]. One of the strategies to overcome MDR is to increase the intracellular concentration of chemotherapeutics using P-glycoprotein inhibitors [15]. Another strategy is to silence the genes involved in MDR using small interfering RNA (siRNA), which seems to be a promising tool.

In our previous work [16], RLS_40 _cell line, exhibiting MDR, was obtained by cultivation of murine ascitic lymphosarcoma RLS cells, resistant to apoptosis-inducing agents, in the presence of vinblastine. The MDR of RLS_40 _cells was provided by a high expression of *mdr1b *and *mdr1a *genes and a moderate expression of *bcl-2 *and appeared as a 20-fold decrease in the cell sensitivity to a wide range of cytostatics (cyclophosphamide, cysplatin, vinblastine, and rubomycin) [16]. The obtained RLS_40 _cell line generates tumors in mice and mimics the tumor status observed in patients after several chemotherapy courses [17]. Thus, we had at our disposal two lymphosarcoma models displaying MDR: the MDR of RLS_40 _is *mdr1b/1a*-associated and the MDR of RLS tumor is predominantly *bcl-2*-associated.

In this study, we applied the mdr1b/1a siRNA targeted to *mdr1b *and *mdr1a *mRNAs and bcl-2 siRNA addressed to *bcl-2 *mRNA aiming to reverse the MDR phenotype of tumor cells and increase their sensitivity to chemotherapeutics. The potential of siRNAs was studied *in vitro*: we showed that mdr1b/1a and bcl-2 siRNAs caused five- and two-fold decrease in the corresponding mRNA levels, respectively, and mdr1b/1a siRNA exerted an increase in cell sensitivity to cytostatics. The application of siRNA followed by cyclophosphamide administration was studied *ex vivo *and *in vivo *using RLS_40 _and the related RLS lymphosarcomas developed in mice. Our data showed that single application of mdr1b/1a siRNA and cyclophosphamide resulted in a decrease in the MDR and a threefold decrease in the tumor size as compared with the control animals treated only with cyclophosphamide, while bcl-2 siRNA was ineffective *in vivo*.

## Methods

### Mice

Male 10-12 week-old CBA/LacSto (hereinafter, CBA) mice obtained from the animal breeding facility with the Institute of Cytology and Genetics (Siberian Branch, Russian Academy of Sciences, Novosibirsk, Russia) were used in this study. All animal procedures were carried out in accordance with approved protocol and recommendations for proper use and care of laboratory animals [European Communities Council Directive 86/609/CEE].

### Tumor generation

Ascites RLS and RLS_40 _lymphosarcomas are permanent and maintained in CBA mice by regular passaging of tumor cells. For generating ascites tumors, 2 × 10^5 ^tumor cells in 0.2 ml of buffered saline were intraperitoneally injected into the abdominal cavities of CBA mice. For generating solid tumors, intramuscular injections of 2 × 10^5 ^tumor cells in 0.1 ml of solution were made into the right lower limbs of CBA mice.

### Isolation of primary tumor culture from ascites

On days 10-12 of RLS or RLS_40 _tumor development, 0.5 ml of phosphate buffered saline (PBS) was injected into the abdominal cavity. Then mice were sacrificed by cervical dislocation to collect the ascites fluid with syringe. To eliminate the erythrocytes, the collected cell suspension was diluted with 5 ml of PBS, layered over 3 ml of LSM (lymphocyte separation medium, MP Biomedicals, United States), and centrifuged at 1500 rpm for 15 min. The tumor cells, localized to the mononuclear fraction, were collected, washed with PBS, and centrifuged at 1000 rpm for 5 min. The collected cells were suspended in the Iscove's Modified Dulbecco's Medium (IMDM) supplemented with 10% fetal bovine serum (FBS), 1% antibiotic antimycotic solution (10 000 μg/ml streptomycin, 10 000 IU/ml penicillin, and 25 μg/ml amphotericin; ICN, Germany). The cells were cultivated at 37°C in a humidified incubator with 5% CO_2 _until a complete adhesion of macrophages to the flask bottom. Then the cell suspension containing lymphocytes was transferred to a new flask and cultivated under the same conditions. In the *in vitro *experiments on analysis of siRNA silencing activity and sensitivity of siRNA-treated tumor cells to cytostatics, the cells were cultivated not more than 1 month. To perform *ex vivo *experiments, the isolated cells were inoculated in mice immediately after transfection with the corresponding siRNA.

### Design of siRNAs

The sequence for mdr1b/1a siRNA was selected using the data from GenBank [GenBank: M14757]; the sequence for bcl-2 siRNA was chosen according to Wacheck *et al*. [18]. The siRNAs had the following sequences: mdr1b/1a siRNA, sense 5'-GGCUGGACAAGCUGUGCAUGG-3' and antisense 5'-AUGCACAGCUUGUCCAGCCAA-3' and bcl-2 siRNA, sense 5'-GCCUUUGUGGAACUAUAUGGdTdT-3' and antisense 5'-CCAUAUAGUUCCACAAAGGCdTdT-3'; luciferase siRNA was used as a control (sense 5'-CGUUAUUUAUCGGAGUUGCAG-3' and antisense 5'-GCAACUCCGAUAAAUAACGCG-3') [19]. The siRNAs were chemically synthesized at the Laboratory of RNA Chemistry (Institute of Chemical Biology and Fundamental Medicine).

### Duplex formation of siRNAs

The sense and antisense strands of siRNAs at a concentration of 20 μM were incubated in annealing buffer (100 mM potassium acetate, 30 mM HEPES-KOH pH 7.4, and 2 mM magnesium acetate) at 90°C for 1 min and then at 37°C for 1 h. The formed duplexes were kept at -20°C.

### Cell transfection with siRNAs

Tumor cells were placed in a serum-free medium into six-well plates immediately before transfection. The cells were incubated with siRNA precomplexed with Lipofectamine™ 2000 (Invitrogen, United States) in an Opti-MEM medium (Invitrogen, United States) at 37°C for 4 h according to the manufacturer's protocol. Then the medium was replaced with a supplemental medium containing 10% FBS, and the cells were cultivated for 24-96 h under standard conditions. In the *ex vivo *experiments, the cells after transfection were twice washed with PBS and diluted with PBS to a concentration of 2 × 10^6 ^cells/ml.

### RNA isolation

Total RNA was isolated from cells according to published protocol [20]. RNA concentration in the samples was measured by absorbance at 260 and 280 nm using a BioMate 3 (Thermo Electron Corporation) spectrophotometer.

### Semiquantitative RT-PCR analysis

The primers and RT-PCR conditions were earlier described in detail [16]. β-Actin product was used as an internal control. Amplification was performed in the following mode: the initial step at 95°C for 5 min; 24, 25, and 30 cycles at 94°C for 1 min, 57°C for 1 min, and 72°C for 1 min for *mdr1b*, *mdr1a*, and *bcl-2*, respectively; and the final elongation step at 72°C for 5 min. The PCR products were analyzed by 8% PAGE, visualized with ethidium bromide staining, photographed in UV light, and densitometrically quantified using a Gel-Pro Analyzer 4.0. The data were presented as the ratio of specific gene expression level to *β-actin *expression level.

### Cell viability in the presence of cytostatics (MTT test)

The change in sensitivity of RLS_40 _cells to vinblastine after transfection with siRNA was tested by MTT assay [21]. Vinblastine (Sigma, United States) was added to cells at concentrations of 10 to 700 nM 48 h after transfection, and the cells were incubated at 37°C for 48 h in a humidified incubator with 5% CO_2_. Then MTT solution was added to cells at a final concentration of 0.5 mg/ml, and the cells were incubated for 3-4 h under the same conditions. The medium was removed; the crystallized formazan was dissolved in 100 μl of DMSO; and optical density was measured at a wavelength of 570 nm (background, at 620 nm) in a Multiscan RS (TermoLabsystems) multichannel spectrophotometer.

### Rhodamine 123 efflux assay

The intracellular accumulation of the substrate of Pgp, Rhodamine 123 (Rh123), was measured to evaluate the Pgp activity in RLS_40 _cells after the transfection with mdr1b/1a siRNA according to [22]. Cells (5 × 10^5^) were washed with PBS, resuspended in 0.5 ml of serum-free IMDM, and incubated with 10 μM of the Pgp inhibitor Verapamil for 45 min at 37°C and 5% CO_2_. To determine the influx, Rh123 was added to cells to a final concentration of 5 μM. The cells were incubated for 15 min at 37°C and 5% CO_2 _in the darkness. After the influx step, the cells were washed with serum-free medium, resuspended in 0.5 ml of this medium, and cultivated for 3 h at 37°C and 5% CO_2 _to determine the efflux. Rh123 efflux was stopped by cooling the cells at 4°C, and the cells were washed with ice-cold PBS. Propidium iodide was added to the cells for assessing viability. The analysis was performed in a Cytomics FC 500 (Beckman Coulter) flow cytometer by accumulating events in the FL1 channel. The median fluorescence (ΔF) of samples was used to calculate the percentage of Rh123 intracellular accumulation (A%):

where ΔF_Ver _is a median fluorescence of the cells incubated with Rh123 and Verapamil; ΔF_mdr1b/1a siRNA_, a median fluorescence of the cells transfected with mdr1b/1a siRNA and incubated with Rh123; and ΔF_luc siRNA_, a median fluorescence of the cells transfected with luciferase siRNA and incubated with Rh123.

### Tumor development in the presence of siRNA and/or cytostatics

*Ex vivo*. RLS_40 _or RLS ascites were taken from CBA mice. The RLS_40 _cells isolated from ascites fluid by filtration through LSM were divided into four portions: (1) wild-type cells (hereinafter, wt cells), (2) the cells transfected with control luciferase siRNA, (3) the cells transfected with mdr1b/1a siRNA at a concentration of 100 nM, and (4) the cells transfected with bcl-2 siRNA at a concentration of 200 nM. The RLS cells isolated from ascites fluid were divided into three portions: (1) wt cells; (2) the cells transfected with control luciferase siRNA; and (3) the cells transfected with bcl-2 siRNA at a concentration of 200 nM. The cell suspensions (0.1 ml, 2 × 10^6 ^cells/ml) 4 h after transfection were intramuscularly inoculated into the right lower limb of 14-16-week-old male CBA mice for solid tumor development. On days 2 and 4 after tumor cell inoculation, a half of the RLS_40_-bearing animals from each group was intraperitoneally injected with 100 mg/kg of cyclophosphamide (Biokhimik, Saransk, Russia) or with 1.5 mg/kg of vinblastine and a half of RLS-bearing animals from each group was once intraperitoneally injected with 200 mg/kg of cyclophosphamide on day 2 after transplantation. The other half of each group was injected with buffered saline. Each group included 40 mice.

*In vivo*. The CBA mice with generated RLS_40 _ascites tumors were divided into two groups: the mice were intraperitoneally injected with (1) 15 μg of control luciferase siRNA or (2) 15 μg of mdr1b/1a siRNA. Prior to injection, the siRNAs were precomplexed with Lipofectamine in Opti-MEM medium. The ascites 4 h after injection were collected from each group of animals, diluted with PBS to a concentration of 2 × 10^6 ^cells/ml, and 0.1 ml of this suspension was intramuscularly inoculated into the right lower limb of 14-16-week-old male mice for solid tumor development. On day 2 after tumor inoculation, each group of animals was divided into three subgroups (20 mice per subgroup): subgroup 1 was intraperitoneally injected with 200 mg/kg of cyclophosphamide; subgroup 2 intravenously injected with 2 mg/kg of embichin (mechlorethamine hydrochloride, Aldrich, United States); and subgroup 3 (control subgroup) was injected with buffered saline.

As soon as tumors began to be palpable, the tumor volumes were measured every 2-3 days using calipers. When the experiment was finished, the animals were sacrificed by cervical dislocation. Tumor weight was determined as the difference between the weights of the amputated tumor-bearing stump and the tumor-free contralateral stump. The inhibition of tumor growth was estimated as follows: [mean tumor weight_control _- mean tumor weight_experiment_]/mean tumor weight_control_. The liver was weighed for hepatic index calculation. Hepatic index was estimated as follows: (liver weight/mouse weight) × 100%. The liver index of healthy CBA mice was 5.0%.

### Statistics

The data were statistically processed using the Student's *t*-test (two-tailed, unpaired); *p *< 0.05 was considered statistically significant.

## Results

### Design of siRNAs

To reverse the MDR phenotype of tumor cells, we applied in our experiments two siRNAs: the bcl-2 siRNA targeted to *bcl-2 *mRNA and the mdr1b/1a siRNA targeted to *mdr1b *and *mdr1a *mRNAs. The mdr1b/1a siRNA was designed to target *mdr1b *and *mdr1a *genes simultaneously. The sequence of mdr1b/1a siRNA antisense strand is complementary to the *mdr1b *mRNA and is able to form a duplex with three mismatches with *mdr1a *mRNA. Note that the mismatches are located at the 5'-end of the antisense strand, which makes this strand favorable for RISC loading [23]. It has been shown earlier that an efficient silencing of *c-myc *and *N-myc *genes is feasible using single siRNA [24]. The sequences of *mdr1b *and *mdr1a *mRNAs for targeting with mdr1b/1a siRNA were chosen based on the results of the studies [25,26] where the siRNAs addressed to various regions of *MDR1 *mRNA were screened: the designed siRNAs displayed silencing activity and no off-target effects.

### *In vitro*

The siRNAs targeted to the mRNA of *mdr1b*, *mdr1a *and *bcl-2 *genes were tested for their ability to downregulate the synthesis of Pgp and Bcl-2 proteins in the RLS_40 _cell line, displaying MDR phenotype. The siRNA targeted to the mRNA of firefly luciferase gene was used as a control. RLS_40 _cells were transfected with specific siRNA at concentrations of 20 to 200 nM in the presence of Lipofectamine and 24-96 h post transfection, the expression levels of *mdr1a*, *mdr1b*, and *bcl-2 *genes were measured by semiquantitative RT-PCR.

**Figure 1:**
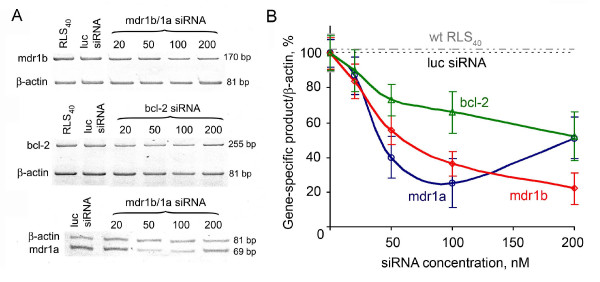
**Silencing of *mdr1b*, *mdr1a*, and *bcl-2 *genes in RLS_40 _cells by siRNAs**. (**A**) RT-PCR analysis (8% PAGE) of *mdr1b*, *bcl-2*, and *mdr1a *mRNA levels in the RLS_40 _cells transfected with control luciferase siRNA, mdr1b/1a siRNA, and bcl-2 siRNA (20-200 nM) 48 h after transfection. (**B**) Dose dependence of the silencing effect of gene-specific siRNAs on *bcl-2*, *mdr1a*, and *mdr1b *genes in RLS_40 _cells 48 h after transfection according to semiquantitative RT-PCR assay. The expression levels of *mdr1b*, *mdr1a*, and *bcl-2 *genes in the tumor cells transfected with luciferase siRNA (specific gene product/*β-actin *= 100%) were used as controls.

According to the kinetics of the gene silencing by specific siRNAs, the minimal levels of *mdr1a*, *mdr1b*, and *bcl-2 *mRNAs in RLS_40 _cell line were detected 48 h after transfection (primary data not shown). At that time point, the bcl-2 and mdr1b/1a siRNAs caused a twofold, fivefold and fourfold reduction in the level of the *bcl-2, mdr1b *and *mdr1a *mRNAs, respectively (Figure 1A, B). However, 96 h after transfection, the levels of mRNAs restored. Analysis of the concentration dependences of gene silencing demonstrated that the bcl-2 and mdr1b/1a siRNAs at a concentration of 200 nM caused a maximal decrease in *bcl-2 *and *mdr1b *mRNA levels (Figure 1A, B). A further increase in mdr1b/1a siRNA concentration up to 200 nM failed to additionally decrease the *mdr1a *mRNA level: the observed change in mRNA levels was within the experimental error.

**Figure 2:**
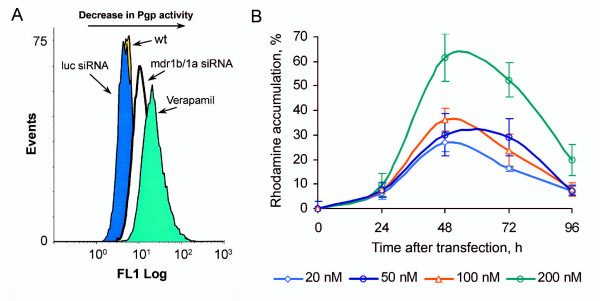
**The levels of Pgp activity in the RLS_40 _cells treated with siRNA according to Rh123 efflux assay**. (**A**) Flow cytometry data showing the inhibitory effect of Verapamil and specific mdr1b/1a siRNA (200 nM, 48 h after transfection). (**B**) Dose and time dependences of the Rh123 accumulation in RLS_40 _cells mediated by *mdr1b *and *mdr1a *gene silencing induced by mdr1b/1a siRNA. The plots show the data of three independent experiments.

The *mdr1a *and *mdr1b *genes of mice encode Pgp, which exports chemotherapeutic agents out of cancer cells, thereby providing for a decrease in drug accumulation in tumors. The silencing effect of mdr1b/1a siRNA on *mdr1a *and *mdr1b *genes should be accompanied by a decrease in Pgp activity; therefore, we studied the effect of mdr1b/1a siRNA on the Pgp pump function. Rh123 is a well-known substrate for Pgp efflux and is commonly used for the measurement of Pgp bioactivity. In the Rh123 efflux assay, the Pgp activity was blocked by a commonly used Pgp inhibitor, Verapamil (as a positive control). Thus, the efficacy of mdr1b/1a siRNA was evaluated relative to the inhibitory effect of Verapamil. Accumulation of Rh123 in the tumor cells cultivated with Verapamil was considered 100%. As expected according to the Rh123 efflux assay, tumor cells intensively pumped out Rh123 in the absence of inhibitors of Pgp activity. The control luciferase siRNA did not influence the Pgp activity (Figure 2A), and Rh123 cellular accumulation was low (regarded as 0%), whereas the efflux of fluorescent dye was considerably decreased in the RLS_40 _cells transfected with mdr1b/1a siRNA (Figure 2A, B). The highest level of Rh123 accumulation was observed 48 and 72 h after transfection with 100 and 200 nM of mdr1b/1a siRNA: the Rh123 accumulation reached 38 and 62%, respectively, as compared with the cells transfected with control siRNA (Figure 2B).

**Figure 3:**
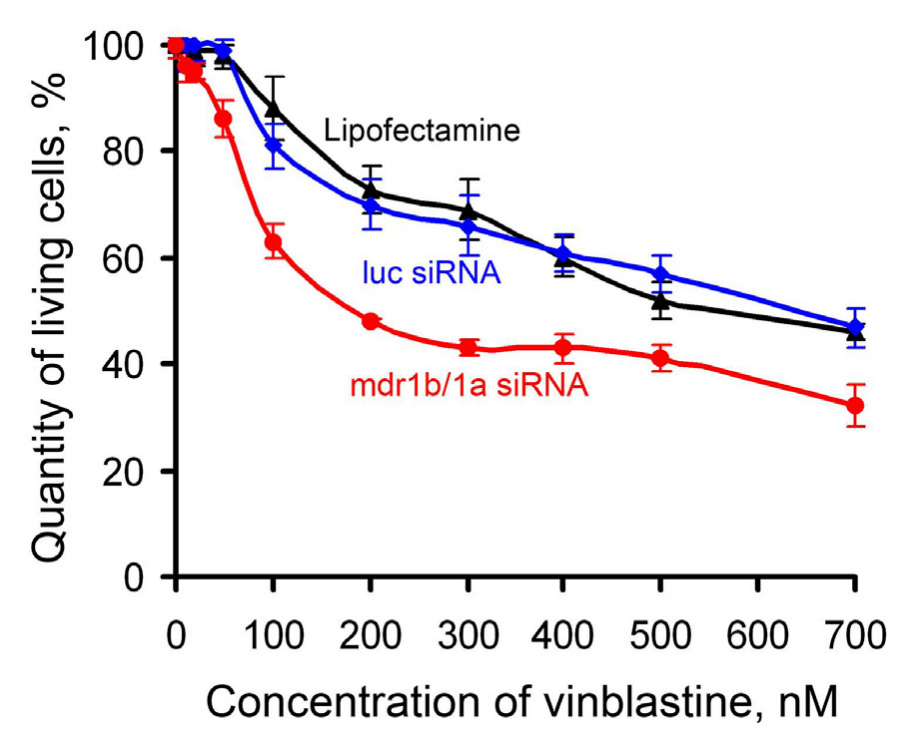
**The sensitivity of RLS_40 _cells to vinblastine**. The viability of RLS_40 _cells transfected with control luciferase siRNA (100 nM) or mdr1b/1a siRNA (100 nM) was measured by MTT-test 48 h after incubation with vinblastine.

The downregulation of *mdr1a *and *mdr1b *gene expression by specific siRNA resulted in a decrease in the Pgp quantity in the cytoplasmic membrane, which contributes to accumulation of cytostatics in the cytoplasm leading to cell death. The sensitivity of RLS_40 _cells to vinblastine was evaluated 48 h after transfection with mdr1b/1a siRNA, when the levels of *mdr1a *and *mdr1b *mRNAs were the lowest. The IC_50 _of vinblastine (concentration of vinblastine at which 50% of the cells remained viable) for the RLS_40 _cells transfected with luciferase siRNA was 691.4 ± 44.2 nM, being similar to the IC_50 _for untransfected RLS_40 _cells. The transfection of RLS_40 _cells with mdr1b/1a siRNA resulted in an up to threefold increase in the cell sensitivity to vinblastine (Figure 3): the IC_50 _of vinblastine was 315.4 ± 25.1, 278.3 ± 23.1, and 235.5 ± 20.1 nM for the cells transfected with 20, 50, and 100 nM of mdr1b/1a siRNA, respectively. Note that for the parental RLS cell line, which is more susceptible to chemotherapy, the IC_50 _value for vinblastine was 6.2 nM.

### *Ex vivo*

The ability of mdr1b/1a and bcl-2 siRNAs to overcome the MDR phenotype was tested in *ex vivo *experiments. The ascites of RLS_40 _or RLS tumors were collected; the cells were isolated and transfected with siRNAs as described in Materials and Methods. Then tumor cells were inoculated into the right thigh muscle of mice for solid tumor development. In the experiment with RLS_40 _tumor, the mice were twice treated with cyclophosphamide (100 mg/kg), on days 2 and 4 after transplantation; in the experiment with parental RLS tumor, the mice were once treated with cyclophosphamide (200 mg/kg) on day 2 after transplantation. After transfection, approximately 5 × 10^5 ^cells from each sample were left and incubated at 37°C and 5% CO_2 _for 48 h. Then the cells were used for measuring of *mdr1a*, *mdr1b*, and *bcl-2 *mRNA levels. RT-PCR analysis demonstrated that mdr1b/1a siRNA caused a fourfold downregulation of expression of *mdr1a *and *mdr1b *mRNAs and bcl-2 siRNA - a twofold downregulation of expression of *bcl-2 *gene as compared with the wt cells and luciferase siRNA-treated cells.

**Figure 4:**
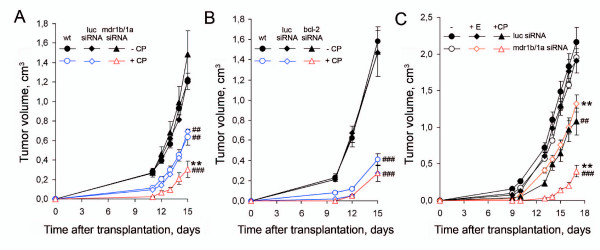
**The dynamics of tumor development in mice after *ex vivo *and *in vivo *successive treatment with siRNA and cytostatics**. (**A**) RLS_40_-bearing animals: **wt**, control mice bearing wild type tumor; **luc siRNA**, the mice bearing tumor cells transfected *ex vivo *with control luciferase siRNA; and **mdr1b/1a siRNA**, the mice bearing tumor cells transfected *ex vivo *with mdr1b/1a siRNA; (**+CP**) the animals treated with cyclophosphamide (100 mg/kg) on days 2 and 4 after tumor transplantation and (**-CP**) the animals without chemotherapeutic treatment. (**B**) RLS-bearing animals: **wt**, the control animals bearing wild type tumor; **luc siRNA**, the mice bearing tumor cells transfected *ex vivo *with control luciferase siRNA; and **bcl-2 siRNA**, the mice bearing tumor cells transfected *ex vivo *with bcl-2 siRNA; (**+CP**) the animals treated with cyclophosphamide (200 mg/kg) on day 2 after tumor transplantation and (**-CP**) the animals without chemotherapeutic treatment. (**C**) RLS_40_-bearing animals: **luc siRNA**, the mice with tumors transfected *in vivo *with luciferase siRNA and **mdr1b/1a siRNA**, the mice with tumors transfected *in vivo *with mdr1b/1a siRNA; (-) the animals without chemotherapeutic treatment, (**+E**) the animals treated with embichin (2 mg/kg) or (**+CP**) cyclophosphamide (200 mg/kg) on day 2 after tumor transplantation. ** *p *< 0.01, compared with cytostatic treatment; ## and ###, p < 0.01 and p < 0.001, respectively, compared with wt (A) or -/luc siRNA and -/mdr1b/1a siRNA (C).

The dynamics of tumor growth is shown in Figure 4. The growth rates of the RLS_40 _tumors either wt or transfected with luciferase or mdr1b/1a siRNA were similar (Figure 4A, black curves), while cyclophosphamide administration resulted in a twofold retardation of tumor growth in the mice with wt RLS_40 _tumors and mice bearing the tumors transfected with luciferase siRNA (Figure 4A, blue curves), thus showing the absence of specific effect of the control siRNA on the tumor growth. The administration of cyclophosphamide in the case of the RLS_40 _cells transfected with mdr1b/1a siRNA resulted in a twofold retardation of tumor growth as compared with only cyclophosphamide action and sixfold, as compared with the untreated tumors (Figure 4A, red curve). The treatment of RLS_40 _cells with the bcl-2 siRNA followed by cyclophosphamide administration had no effect on the RLS_40 _tumor growth (data not shown), which is explainable by a major contribution of *mdr1a *and *mdr1b *gene products to MDR development (Pgp-mediated MDR phenotype) as compared with *bcl-2 *gene.

The block of apoptosis is a distinctive feature of the parental RLS tumor: a sixfold increase in the expression level of *bcl-2 *mRNA was observed for the parental RLS lymphosarcoma as compared with RLS_40 _tumor. Therefore, an antitumor potential of bcl-2 siRNA and cyclophosphamide was examined *ex vivo *using the RLS lymphosarcoma. Analysis of dynamics of RLS tumor growth failed to find any significant difference between the growth rates of wt tumor, the tumor transfected with control luciferase siRNA, and specific bcl-2 siRNA after cyclophosphamide impact (Figure 4B).

The ability of mdr1b/1a siRNA to sensitize RLS_40 _tumor to cytostatics was also studied in an *ex vivo *experiment using vinblastine. The dynamics of tumor growth showed that the double treatment including mdr1b/1a siRNA and vinblastine had no effect on tumor progress (data not shown). The reason could be a high resistance of RLS_40 _tumor cells to this particular cytostatic (IC_50 _= 680 ± 42 nM): the tolerable dose of vinblastine administered to animals was lower than the IC_50 _values observed *in vitro *even after silencing of *mdr1b *gene with the corresponding siRNA (283 ± 4 nM).

In all groups of mice, the hepatic index (HI), an indicator of animal health, was measured. The HI in healthy CBA mice is 5.0%. In the groups of the RLS_40_-bearing animals treated with cyclophosphamide only or cyclophosphamide following the transfection with the luciferase siRNA, the HI was 8.6 ± 0.6%. The HI was 6.1 ± 0.9% in the mice bearing RLS_40 _tumors treated with cyclophosphamide after mdr1b/1a siRNA transfection. An average lifetime for mice of this group was 25 ± 1.8 days versus 21.4 ± 1.5 days in the groups treated with cyclophosphamide only or cyclophosphamide with the control siRNA.

### *In vivo*

**Figure 5:**
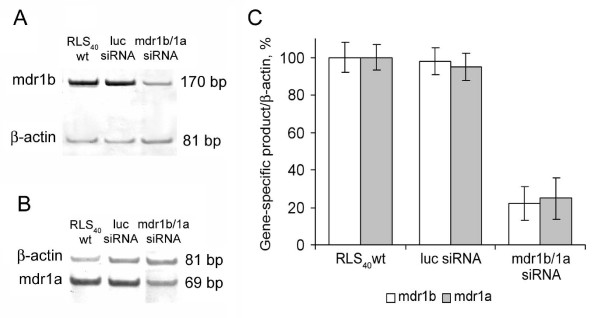
**Silencing of *mdr1b *and *mdr1a *genes in lymphosarcoma RLS_40 _by siRNAs**. RT-PCR analysis (8% PAGE) of *mdr1b *mRNA level (**A**) and *mdr1a *mRNA level (**B**) 48 h after transfection of RLS_40 _ascites with 15 μg of luciferase or mdr1b/1a siRNA *in vivo*. (**C**) The expression levels of *mdr1b *and *mdr1a *genes in the tumor cells transfected with *mdr1b/1a *siRNA. The expression levels of *mdr1b *and *mdr1a *genes in the wild-type tumor cells and cells transfected with luc siRNA were used as controls.

The efficacy of cytostatic treatment following the silencing of *mdr1b *and *mdr1a *genes with siRNA was tested *in vivo*. RLS_40 _ascites tumors were transfected *in vivo *with (1) luciferase siRNA or (2) mdr1b/1a siRNA. The RLS_40 _ascites were extracted 4 h post transfection, and the cells were intramuscularly transplanted into new groups of mice for solid tumor formation. After transfection, approximately 10^6 ^cells were isolated from the ascites fluid and incubated at 37°C and 5% CO_2 _for 48 h. Then the cells were used for measuring the *mdr1a *and *mdr1b *mRNA levels. RT-PCR analysis demonstrated that the mdr1b/1a siRNA caused a fourfold downregulation of expression of *mdr1a *and *mdr1b *genes as compared with the luciferase siRNA-treated cells (Figure 5).

On day 2 of tumor development, the mice were treated with embichin (2 mg/kg) or cyclophosphamide (200 mg/kg). Analysis of the tumor growth dynamics showed that the tumors derived from the cells transfected with mdr1b/1a siRNA were more sensitive to chemotherapy as compared with the cells transfected with the control siRNA (Figure 4C). Both cytostatics, embichin and cyclophosphamide, caused a significant retardation of tumor growth only in the mice treated with mdr1b/1a siRNA: the tumors were 1.4-fold smaller in the case of embichin (Figure 4C, orange curve) and 3.3-fold smaller in the case of cyclophosphamide (Figure 4C, red curve) as compared with the corresponding control groups. On the other hand, administration of embichin only did not influence the tumor growth in the control group (the tumors transfected with luciferase siRNA) (Figure 4C, black curve, diamond points). Cyclophosphamide had a more pronounced effect on the tumor growth: the tumors transfected with luciferase siRNA were twofold smaller (Figure 4C, black curve, triangle points) than the tumors without treatment. The HI in the group received luciferase siRNA and cyclophosphamide was 9.2 ± 0.7% versus 7.7 ± 0.8% in the group received mdr1b/1a siRNA. An average lifetime for the mice of this group was 20.6 ± 0.7 days versus 16.8 ± 0.5 days in the group treated with cyclophosphamide only or cyclophosphamide with the control siRNA.

## Discussion

The range of siRNA application continues to expand. Researchers attempt to explore the potential of RNA interference (RNAi) technology for the therapy of communicable, autoimmune, cancer, and inflammatory diseases; neural disorders; chronic pains; adiposis; and insulin-independent diabetes [27-34]. The potential of siRNA strategy to overcome multidrug resistance *in vitro *was demonstrated in a number of studies [25,26,35-39], and the success in *in vivo *experiments is retarded by unsettled challenges in the *in vivo *siRNA delivery technologies [40,41].

Development of MDR to a variety of conventional and novel chemotherapeutic agents is a significant obstacle in effective therapy of refractory cancer types. In our previous work, we established two murine lymphosarcoma models with a stable MDR, namely, RLS and RLS_40 _[16]. These two related tumors differed in the origin of drug resistance: the MDR of RLS_40 _tumor is *mdr1a*/*mdr1b*-associated and the MDR of RLS tumor is *bcl-2*-associated. These lymphosarcomas are the allografts, exhibiting the adequate response of the body.

In this work, we applied the siRNA targeted to the mRNAs of *bcl-2, mdr1b*, and *mdr1a *genes to overcome the MDR phenotype of lymphosarcomas RLS_40 _and RLS. It was shown that mdr1b/1a siRNA caused the decrease in both *mdr1b *and *mdr1a *mRNA levels. Although the mdr1b/1a siRNA has three mismatches in the binding site of *mdr1a *mRNA, we believe that this siRNA does not provide a miRNA effect and acts via the RNA interference mechanism. The key features that distinguish a miRNA from a siRNA are (1) a noncomplementarity between the center on the miRNA and the targeted mRNA [42,43] and (2) a decrease in the protein level rather than the mRNA level [44]. In our case, we observed a concentration-dependent reduction in the *mdr1a *mRNA level after treating the cells with mdr1b/1a siRNA. The mismatches between mdr1b/1a siRNA and *mdr1a *mRNA are localized to the 5'-end of the siRNA antisense strand rather than to its central part. Therefore, the total effect of MDR reversing is provided by the degradation of both *mdr1b *and *mdr1a *mRNAs via an RNA-interference pathway.

The effect of mdr1b/1a siRNA resulted in a substantial increase in the sensitivity of cancer cells to chemotherapeutics both *in vitro *and *in vivo *and led to a threefold retardation of tumor growth upon cyclophosphamide administration. Interestingly, we observed a more efficient inhibition of tumor growth in *in vivo *experiment as compared with *ex vivo *experiment. It could be explained by the difference in the treatment schemes, namely, a double administration of cyclophosphamide on days 2 and 4 after tumor implantation at a dose of 100 mg/kg in the *ex vivo *experiment and a single administration of cyclophosphamide on day 2 at a doubled dose in the *in vivo *experiment.

Our data demonstrated that the mdr1b/1a siRNA *in vivo *sensitized RLS_40 _tumor cells to cyclophosphamide and embichin. Cyclophosphamide itself is not cytotoxic but undergoes metabolic changes in the body predominantly in the liver with the formation of an active derivative, phosphoramide mustard [45], which is an alkylating agent capable of forming cross-links in DNA molecules and initiating apoptosis. Phosphoramide mustard has not been regarded as a substrate of Pgp [46]. The observed inhibition of RLS_40 _tumor growth after an *ex vivo *treatment of tumor cells with mdr1b/1a siRNA followed by *in vivo *administration of cyclophosphamide suggests that this toxic agent is yet a substrate of Pgp. To confirm this assumption in *in vivo *experiment, mice were injected with embichin (nitrogen mustard), another active alkylating agent with similar cytotoxic properties. Although embichin was not as efficient as cyclophosphamide, the obtained results supported this hypothesis.

Our data showed that bcl-2 siRNA specifically downregulated the *bcl-2 *mRNA level by 50% *in vitro*; however, the bcl-2 siRNA *in vivo *neither showed any sensitizing effect on RLS tumor nor influenced the tumor growth. In addition to the classical Pgp-based mechanism of multidrug resistance of cancer cells [47-50], there is an alternative mechanism associated with putative effector pathways of drug action, apoptosis, and senescence [51-59]. It was shown that Pgp could modulate caspase activation, e.g., caspase 8, and inhibit apoptosis [60]. Thus, the Pgp inhibitors, which reverse drug resistance, can also block its caspase-inhibitory function [60]. Although an MDR phenotype of RLS is considered as *bcl-2*-associated, the expression levels of *mdr1a *and *mdr1b *genes in these cells are also rather high [16]. In both RLS and RLS_40 _cells, high levels of Pgp and Bcl-2 resulted in a "double" suppression of apoptosis. The mdr1b/1a siRNA-induced decrease in the Pgp level resulted in triggering of proapoptosis, while the decrease in Bcl-2 caused by bcl-2 siRNA is not sufficient to overcome the MDR of tumor due to a high Pgp level. Another possible reason for the absence of tumor sensitivity to cyclophosphamide after bcl-2 siRNA transfection is that a 50% downregulation of *bcl-2 *mRNA expression can be insufficient.

The results demonstrating the sensitizing properties of mdr1b/1a siRNA cannot be immediately applied in the clinical practice; however, it is really encouraging that even a single administration of siRNA resulted in essential tumor growth retardation and increase in the average lifetime by approximately 20%. Thus, the examined therapy has significant promise in the clinical management of refractory cancer diseases.

## Conclusions

In view of our results, the synthetic siRNA targeted to *mdr1b *and *mdr1a *genes overcame MDR phenotype *in vivo *and, in the successive treatment with conventionally used cytostatics, evoked more than threefold increase in the tumor cell sensitivity to chemotherapy after a single siRNA administration. In clinical practice, a twofold decrease in the cancer cell resistance to chemotherapy is sufficient to achieve a significant enhancement in the efficiency of antitumor therapy.

## Competing interests

The authors declare that they have no competing interests.

## Authors' contributions

OP performed most of the experiments and data analysis and drafted the manuscript. NM coordinated the study and participated in the experiments and manuscript preparation. NP participated in the design and coordination of the animal study. VK participated in the animal study and revision of the manuscript. VN participated in the animal study and data interpretation. VV conceived the study and revised the manuscript. MZ developed the experimental concept and design and supervised the study, data interpretation, and manuscript revision.

All authors read and approved the final manuscript.

## Pre-publication history

The pre-publication history for this paper can be accessed here:

http://www.biomedcentral.com/1471-2407/10/204/prepub

## References

[B1] Ambudkar S Dey S Hrycyna C Ramachandra M Pastan I Gottesman MM Biochemical, cellular and pharmacological aspects of the multidrug transporter Annu Rev Pharmacol Toxicol 1999 39 361 398 10.1146/annurev.pharmtox.39.1.361 10331089

[B2] Gottesman MM Mechanisms of cancer drug resistance Annu Rev Med 2002 53 615 627 10.1146/annurev.med.53.082901.103929 11818492

[B3] Choi CH ABC transporters as multidrug resistance mechanisms and the development of chemosensitizers for their reversal Cancer Cell Int 2005 5 30 10.1186/1475-2867-5-30 16202168 PMC1277830

[B4] Ullah MF Cancer Multidrug Resistance (MDR): A Major Impediment to Effective Chemotherapy Asian Pacific J Cancer Prev 2008 9 1 6 18439063

[B5] Scotto KW Transcriptional regulation of ABC drug transporters Oncogene 2003 22 7496 7511 10.1038/sj.onc.1206950 14576854

[B6] Stavrovskaya AA Cellular mechanisms of multidrug resistance of tumor cells Biochemistry (Mosc) 2000 65 95 106 10702644

[B7] Stavrovskaya AA Stromskaya TP Transport proteins of the ABC family and multidrug resistance of tumor cells Biochemistry (Mosc) 2008 73 592 604 10.1134/S0006297908050118 18605983

[B8] Galimberti S Marchetti A Buttitta F Carnicelli V Pellegrini S Bevilacqua G Petrini M Multidrug resistance related genes and p53 expression in human non small cell lung cancer Anticancer Res 1998 18 2973 2976 9713494

[B9] Breen L Heenan M Amberger-Murphy V Clynes M Investigation of the role of p53 in chemotherapy resistance of lung cancer cell lines Anticancer Res 2007 27 1361 1364 17593631

[B10] Maung ZT MacLean FR Reid MM Pearson AD Proctor SJ Hamilton PJ Hall AG The relationship between *bcl-2 *expression and response to chemotherapy in acute leukaemia Br J Haematol 1994 88 105 109 10.1111/j.1365-2141.1994.tb04984.x 7803231

[B11] Ishikawa H Kawano MM Okada K Tanaka H Tanabe O Sakai A Asaoku H Iwato K Nobuyoshi M Kuramoto A Expressions of DNA topoisomerase I and II gene and the genes possibly related to drug resistance in human myeloma cells Br J Haematol 1993 83 68 74 10.1111/j.1365-2141.1993.tb04633.x 8094626

[B12] Honjo K Takahashi KA Mazda O Kishida T Shinya M Tokunaga D Arai Y Inoue A Hiraoka N Imanishi J Kubo T MDR1a/1b gene silencing enhances drug sensitivity in rat fibroblast-like synoviocytes J Gene Med 2010 12 219 227 19950109 10.1002/jgm.1378

[B13] Zhang F Riley J Gant TW Intrinsic multidrug class 1 and 2 gene expression and localization in rat and human mammary tumors Lab Invest 1996 75 413 426 8804364

[B14] Borst P Jonkers J Rottenberg S What Makes Tumors Multidrug Resistant? Cell Cycle 2007 6 2782 2787 17998803 10.4161/cc.6.22.4936

[B15] Shukla S Wu CP Ambudkar SV Development of inhibitors of ATP-binding cassette drug transporters - present status and challenges Expert Opin Drug Metab Toxicol 2008 4 205 223 10.1517/17425255.4.2.205 18248313

[B16] Mironova N Shklyaeva O Andreeva E Popova N Kaledin V Nikolin V Vlassov V Zenkova M Animal model of drug-resistant tumor progression Ann NY Acad Sci 2006 1091 490 500 10.1196/annals.1378.090 17341638

[B17] Zenkov AN Scvortsova NV Chernolovskaya EL Pospelova TI Vlassov VV Expression of the MDR1 and MRP genes in patients with lymphoma with primary bone marrow involvement Nucleosides Nucleotides Nucleic Acids 2004 23 843 847 10.1081/NCN-200026029 15560070

[B18] Wacheck V Losert D Gunsberg P Vornlocher HP Hadwiger P Geick A Pehamberger H Muller M Jansen B Small interfering RNA targeting *bcl-2 *sensitizes malignant melanoma Oligonucleotides 2003 13 393 400 10.1089/154545703322617078 15000830

[B19] Elbashir SM Harborth J Lendeckel W Yalcin A Weber K Tuschl T Duplexes of 21-nucleotide RNAs mediate RNA interference in cultured mammalian cells Nature 2001 411 494 498 10.1038/35078107 11373684

[B20] Chattopadhyay N Kher R Godbole M Inexpensive SDS/phenol method for RNA extraction from tissues Biotechniques 1993 15 24 26 7689850

[B21] Park JG Kramer BS Steinberg SM Carmichael J Collins JM Minna JD Gazdar AF Chemosensitivity testing of human colorectal carcinoma cell lines using a tetrazolium-based colorimetric assay Cancer Res 1987 47 5875 5879 3664487

[B22] Mazzanti R Gatmaitan Z Croop J Shu H Arias I Quantitative image analysis of rhodamine 123 transport by Adriamycin-sensitive and resistant NIH 3T3 and human hepatocellular carcinoma (Alexander) cells J Cell Pharmacol 1990 1 50 56

[B23] Khvorova A Reynolds A Jayasena SD Functional siRNAs and miRNAs exhibit strand bias Cell 2003 115 209 216 10.1016/S0092-8674(03)00801-8 14567918

[B24] Kabilova TO Vladimirova AV Chernolovskaya EL Vlassov VV Arrest of cancer cell proliferation by dsRNAs Ann NY Acad Sci 2006 1091 425 436 10.1196/annals.1378.085 17341633

[B25] Logashenko EB Vladimirova AV Repkova MN Venyaminova AG Chernolovskaya EL Vlassov VV Silencing of MDR 1 gene in cancer cells by siRNA Nucleosides Nucleotides Nucleic Acids 2004 23 861 866 10.1081/NCN-200026032 15560073

[B26] Logashenko EB Vladimirova AV Zenkov AN Repkova MN Ven'yaminova AG Chernolovskaya EL Vlassov VV Reversion of the multiple-drug resistance phenotype mediated by short interfering RNAs Russ Chem Bull 2005 5 1260

[B27] Ralph GS Radcliffe PA Day DM Carthy JM Leroux MA Lee DC Wong LF Bilsland LG Greensmith L Kingsman SM Mitrophanous KA Mazarakis ND Azzouz M Silencing mutant SOD1 using RNAi protects against neurodegeneration and extends survival in an ALS model Nat Med 2005 11 429 433 10.1038/nm1205 15768029

[B28] Nakamura H Siddiqui SS Shen X Malik AB Pulido JS Kumar NM Yue BY RNA interference targeting transforming growth factor-beta type II receptor suppresses ocular inflammation and fibrosis Mol Vis 2004 4 703 711 15475878

[B29] Ponnappa BC siRNA for inflammatory diseases Curr Opin Investig Drugs 2009 10 418 424 19431074

[B30] Aouadi M Tesz GJ Nicoloro SM Wang M Chouinard M Soto E Ostroff GR Czech MP Orally delivered siRNA targeting macrophage Map4k4 suppresses systemic inflammation Nature 2009 458 1180 1184 10.1038/nature07774 19407801 PMC2879154

[B31] Schiffelers RM Xu J Storm G Woodle MC Scaria PV Effects of treatment with small interfering RNA on joint inflammation in mice with collagen-induced arthritis Arthritis Rheum 2005 52 1314 1318 10.1002/art.20975 15818667

[B32] Zhang X Shan P Jiang D Noble PW Abraham NG Kappas A Lee PJ Small interfering RNA targeting heme oxygenase-1 enhances ischemia-reperfusion-induced lung apoptosis J Biol Chem 2004 279 10677 10684 10.1074/jbc.M312941200 14688267

[B33] Makimura H Mizuno TM Mastaitis JW Agami R Mobbs CV Reducing hypothalamic AGRP by RNA interference increases metabolic rate and decreases body weight without influencing food intake BMC Neurosci 2002 3 18 10.1186/1471-2202-3-18 12423556 PMC134599

[B34] Jun H Song Z Chen W Zanhua R Yonghong S Shuxia L Huijun D *In vivo *and *in vitro *effects of SREBP-1 on diabetic renal tubular lipid accumulation and RNAi-mediated gene silencing study Histochem Cell Biol 2009 131 327 345 10.1007/s00418-008-0528-2 19048273

[B35] Nieth C Priebsch A Stege A Lage H Modulation of the classical multidrug resistance (MDR) phenotype by RNA interference (RNAi) FEBS Lett 2003 545 144 150 10.1016/S0014-5793(03)00523-4 12804765

[B36] Duan Z Brakora KA Seiden MV Inhibition of ABCB1 (MDR1) and ABCB4 (MDR3) expression by small interfering RNA and reversal of paclitaxel resistance in human ovarian cancer cells Mol Cancer Ther 2004 3 833 838 15252144

[B37] Wu C-P Calcagno AM Ambudkar SV Reversal of ABC drug transporter-mediated multidrug resistance in cancer cells: Evaluation of current strategies Curr Mol Pharmacol 2008 1 93 105 10.2174/1874467210801020093 19079736 PMC2600768

[B38] Stierle V Laigle A Jolles B Modulation of MDR1 gene expression in multidrug resistant MCF7 cells by low concentrations of small interfering RNAs Biochem Pharmacol 2005 70 1424 1430 16214115 10.1016/j.bcp.2005.08.007

[B39] Widmer N Rumpold H Untergasser G Fayet A Buclin T Decosterd LA Resistance reversal by RNAi silencing of MDR1 in CML cells associated with increase in imatinib intracellular levels Leukemia 2007 21 1561 1562 10.1038/sj.leu.2404671 17429432

[B40] Oh YK Park TG siRNA delivery systems for cancer treatment Adv Drug Deliv Rev 2009 61 850 862 10.1016/j.addr.2009.04.018 19422869

[B41] Gondi CS Rao JS Concepts in *in vivo *siRNA delivery for cancer therapy J Cell Physiol 2009 220 285 291 10.1002/jcp.21790 19391103 PMC2692765

[B42] Doench JG Petersen CP Sharp PA siRNAs can function as miRNAs Genes Dev 2003 17 438 42 10.1101/gad.1064703 12600936 PMC195999

[B43] Zeng Y Wagner EJ Cullen BR Both natural and designed micro RNAs can inhibit the expression of cognate mRNAs when expressed in human cells Molecular Cell 2002 9 1327 1333 10.1016/S1097-2765(02)00541-5 12086629

[B44] Olsen PH Ambros V The lin-4 regulatory RNA controls developmental timing in C. elegans by blocking LIN-14 protein synthesis after the initiation of translation Develop Biol 1999 216 671 680 10.1006/dbio.1999.9523 10642801

[B45] Boddy AV Yule SM Metabolism and pharmacokinetics of oxazaphosphorines Clin Pharmacokinet 2000 38 291 304 10.2165/00003088-200038040-00001 10803453

[B46] Sakaeda T Nakamura T Okumura K MDR1 genotype-related pharmacokinetics and pharmacodynamics Biol Pharm Bull 2002 25 1391 1400 10.1248/bpb.25.1391 12419946

[B47] Juliano RL Ling V A surface glycoprotein modulating drug permeability in Chinese hamster ovary cell mutants Biochim Biophys Acta 1976 455 152 162 10.1016/0005-2736(76)90160-7 990323

[B48] Borst P Oude Elferink R Richardson CC, Kornberg R, Raetz CHR, Thorstensen K Mammalian ABC transporters in health and disease Ann Rev Biochem 2002 California: Science 537 592 10.1146/annurev.biochem.71.102301.093055 12045106

[B49] Gottesman MM Fojo T Bates SE Multidrug resistance in cancer: Role of ATP-dependent transporters Nat Rev Cancer 2002 2 48 58 10.1038/nrc706 11902585

[B50] Sarkadi B Homolya L Szakacs G Varadi A Human multidrug resistance ABCB and ABCG transporters: Participation in a chemoimmunity defense system Physiol Rev 2006 86 1179 1236 10.1152/physrev.00037.2005 17015488

[B51] Lowe SW Bodis S McClatchey A Remington L Earl Ruley H Fisher DE Housman DE Jacks T p53 Status and the efficacy of cancer therapy in vivo Science 1994 266 807 810 10.1126/science.7973635 7973635

[B52] Schmitt CA Fridman JS Yang M Lee S Baranov E Hoffman RM Lowe SW A senescence program controlled by p53 and p16INK4a contributes to the outcome of cancer therapy Cell 2002 109 335 346 10.1016/S0092-8674(02)00734-1 12015983

[B53] Schmitt CA Lowe SW Apoptosis is critical for drug response in vivo Drug Resist Updat 2001 4 132 134 10.1054/drup.2001.0188 11512522

[B54] Schmitt CA Wallace-Brodeur RR Rosenthal CT McCurrach ME Lowe SW DNA damage responses and chemosensitivity in the E mu-myc mouse lymphoma model Cold Spring Harb Symp Quant Biol 2000 65 499 510 10.1101/sqb.2000.65.499 12760067

[B55] Schmitt CA Rosenthal CT Lowe SW Genetic analysis of chemoresistance in primary murine lymphomas Nature Med 2000 6 1029 1035 10.1038/79542 10973324

[B56] Schmitt CA McCurrach ME de Stanchina E Wallace-Brodeur RR Lowe SW *INK4a/ARF *mutations accelerate lymphomagenesis and promote chemoresistance by disabling p53 Genes and Dev 1999 13 2670 2677 10.1101/gad.13.20.2670 10541553 PMC317110

[B57] Johnstone RW Ruefli AA Lowe SW Apoptosis: A link between cancer genetics and chemotherapy Cell 2002 108 153 164 10.1016/S0092-8674(02)00625-6 11832206

[B58] Schmitt CA Senescence, apoptosis and therapy-cutting the lifelines of cancer Nat Rev Cancer 2003 3 286 295 10.1038/nrc1044 12671667

[B59] Robinson U Roberts WK Ling TT Lamming D Sternberg SS Roepe PD Human MDRl protein overexpression delays the apoptotic cascade in Chinese hamster ovary fibroblasts Biochemistry 1997 36 11169 11178 10.1021/bi9627830 9287159

[B60] Johnstone RW Ruefli AA Tainton KM Smyth MJ A role for P-glycoprotein in regulating cell death Leukemia and Lymphoma 2000 38 1 11 10811443 10.3109/10428190009060314

